# Cardiac safety of ribociclib evaluated with 24-hour rhythm Holter electrocardiogram

**DOI:** 10.1007/s00520-024-08702-0

**Published:** 2024-07-08

**Authors:** Ayse Irem Yasin, Mahmut Uluganyan, Zehra Sucuoglu Isleyen, Atakan Topcu, Abdallah Tm Shbair, Melih Simsek, Mehmet Besiroglu, Yeliz Emine Ersoy, Hacı Mehmet Türk, Mesut Seker

**Affiliations:** 1 Department of Medical Oncology, Bezmialem Vakıf University, Huzur Mah. Cumhuriyet Cad., 34396 Istanbul, Turkey; 2 Department of Cardiology, Bezmialem Vakıf University, Huzur Mah. Cumhuriyet Cad., 34396 Istanbul, Turkey; 3 Department of General Surgery, Bezmialem Vakıf University, Huzur Mah. Cumhuriyet Cad. , 34396 Istanbul, Turkey

**Keywords:** Cardiotoxicity, Holter ECG, Hormon-positive breast cancer, Ribociclib

## Abstract

**Objective:**

We aimed to evaluate cardiac safety profile of ribociclib with 24-h rhythm Holter ECG.

**Material and method:**

Forty-two female metastatic breast cancer patients were included in the study. Rhythm Holter ECG was performed before starting treatment with ribociclib and after 3 months of the treatment initiation.

**Results:**

The mean age of the patients was 56.36 ± 12.73. 52.4% (*n* = 22) of the patients were using ribociclib in combination with fulvestrant and 47.6% (*n* = 20) with aromatase inhibitors. None of the patients developed cardiotoxicity. When the rhythm Holter results before and in third month of the treatment were compared, there was no statistically significant difference.

**Conclusion:**

This is the first study evaluating effects of ribociclib treatment on cardiac rhythm with Holter ECG. The findings suggested ribociclib has a low risk of causing early cardiotoxicity.

## Introduction

Breast cancer is the most common cancer in women worldwide. There are three main molecular subtypes of the breast cancer, which are guiding to choose the best treatment option for the patients: hormone receptor–positive breast cancer, HER2-positive breast cancer, and triple negative breast cancer. Breast cancer treatment is multidisciplinary and planned according to the stage, histology, and molecular subtype of the cancer. For early stage diseases, surgery-based treatment is the standard. Postoperative management strategies included chemotherapy, radiotherapy, hormonotherapy, targeted therapy, and immunotherapy. Locally advanced breast cancers are mostly treated with preoperative/neoadjuvant chemotherapy. Neoadjuvant hormonotherapy for hormone receptor–positive breast cancer and chemo-immunotherapy combinations for triple negative breast cancer are also possible options. For metastatic breast cancer patients, systemic treatment with chemotherapy, immunotherapy, targeted therapy, or hormonotherapy remains the preferred option; surgery is performed only for palliation in selected cases [1–3].

It is known that about three quarters of all breast cancers are hormone receptor–positive, which is a prognostic and predictive factor for endocrine treatment response [1]. Hormonotherapies in breast cancer aim to prevent tumor growth by reducing circulating estrogen or antagonizing the effect of estrogen at the tissue level [2].

Current therapeutic options for metastatic hormone receptor–positive HER2 negative breast cancer patients without visceral crisis include selective ER modulators (SERMs), selective ER degraders (SERDs), aromatase inhibitors (AIs), and cyclin-dependent kinase (CDK) 4/6 inhibitors [3].

There are three CDK 4/6 inhibitors approved by the FDA: abemaciclib, palbociclib, and ribociclib. CDK inhibitors which are selective for CDK 4 and 6 arrest the cell cycle in the G1 phase by inhibiting retinoblastoma protein phosphorylation. It is known that this effect is further increased when used in combination with endocrine agents [4–7]. Ribociclib from this group of drugs, which contributes significantly to PFS in hormone-sensitive metastatic breast cancer, is the only CDK4/6 inhibitor with overall survival (OS) contribution in addition to PFS [8]. Furthermore, interim analyses of the NATALEE study showed longer iDFS in the high-risk stage 2–3 hormone-positive breast cancer patients treated with ribociclib in the adjuvant setting [9]. Despite remarkable efficacy, potential cardiac toxicities remain a concern. Advanced breast cancer patients may have cardiac dysfunction due to prior cardiotoxic chemotherapies. In a recent meta-analysis, ribociclib was found to be associated with a significantly higher risk of QTc prolongation and the resultant dosing inconsistencies and discontinuation [10].

Ribociclib is the only CDK 4/6 inhibitor with a warning for QT prolongation in the FDA label [11]. Therefore, it is recommended to monitor ECG before treatment initiation and not to start ribociclib on patients with QTcF ≥ 450 ms. The ECG should be repeated on day 14 of the first cycle and at the beginning of the second cycle. If QTcF is > 500 ms on ECG treatment should be interrupted until QTcF resolves to < 481 ms. The treatment may resume at the next lower dose level. The QTcF values greater than 500 ms or any increase greater than 60 ms or serious cardiac adverse effects are indications of permanent discontinuation of ribociclib [12].

A recent real-world study showed 24% of the patients experienced CVAEs, which is a higher frequency than previous phase 3 trials among breast cancer patients using CDK4/6 inhibitors reported. Furthermore, they found that cardiac adverse effects of CDK4/6 inhibitors are not limited to QT prolongation and the median time was 2.3 months for developing CVAEs [13]. Most common CVAEs were hypertension (16%), cardiomyopathy/heart failure (5.8%), AF/AFL (4.9%), ischemic heart disease (3.5%), and pericardial disease (2%) [13].

In this study, we aimed to investigate whether a more detailed rhythm evaluation is required to address concerns about the cardiotoxicity that ribociclib may cause. While instantaneous rhythm disturbances are detected by ECG, 24-hour rhythm Holter can provide more accurate information about the effect of the drug on rhythm. We evaluated the effect of the drug on cardiac rhythm in more detail by recording the changes in rhythm Holter at the beginning of treatment. We repeated the rhythm Holter in the third month of the treatment because the routine recommendation for ECG monitoring is only in the first month, and recent studies demonstrated cardiac adverse events were mostly developed in the first 3 months of the treatment [13].

## Material and method

Patients followed up in Bezmialem Vakif University Medical Oncology clinic between June 2021 and June 2022 were screened. Fourty-two female metastatic breast cancer patients using ribociclib combined with any endocrine therapy were enrolled in the study. Patient characteristics, clinical features, and comorbidities of the patients were recorded. An echocardiogram and rhythm Holter ECG were performed before starting treatment with ribociclib and after 3 months of the treatment initiation. A SEER 12 digital Holter ECG recorder was connected to each patient. Patients were allowed to continue to their daily activities. The 24-h Holter recording was then transferred to a computer-based ECG analysis software. We collected baseline rhythm, heart rates (average/minimum/maximum), and all types of ventricular and supraventricular arrhythmias. Transthoracic echocardiography was performed using a Vivid 7 system (GE Vingmed Ultrasound AS, Horten, Norway). LV end-diastolic volume (EDV), end-systolic volume (ESV), left atrial volume, and EF were assessed. QTcF was calculated according to Friderica formula [14]. QT values were recorded before treatment initiation, on day 14 of the cycle 1, at the beginning of cycles 2 and 3. Electrolyte abnormalities, hemoglobin levels, and thyroid functions of the patients were checked to exclude other factors causing cardiac arrhythmia. Patients with known cardiac arrhythmia, ischemic heart disease, diseases causing QT prolongation, or using QT prolonging drugs were excluded. Patients signed an informed consent form before enrollment. The study complied in accordance with the Helsinki Declaration and approved by the Bezmialem Vakıf University ethic approval committee (2020/7–30).

### Statistical analysis

Statistical analysis was performed using the IBM® SPSS 24.0 program; for continuous variables, mean values were taken, and for categorical variables, number and percentage ratios were used. Kolmogorov–Smirnov test was used for normal distribution. Qualitative variables were compared using the Pearson *χ*^2^ test. For repeated variables, Friedman’s ANOVA test was used. The characteristics of patients were evaluated with descriptive analysis. Statistical significance was accepted as *p* < 0.05.

## Results

The mean age of the patients was 56.36 ± 12.73, and 50% (*n* = 21) of them had de novo metastatic disease. 52.4% (*n* = 22) of the patients were using ribociclib in combination with fulvestrant and 47.6% (*n* = 20) with aromatase inhibitors. 26.2% (*n* = 11) of the patients were using tobacco, 14.3% (*n* = 6) had diabetes mellitus, and 31% (*n* = 13) had hypertension. 61.9% (*n* = 26) of the patients experienced grade 1–2 neutropenia, and 28.5% (*n* = 12) had grade 3 neutropenia requiring dose reduction. 9.5% (*n* = 4) of the patients had grade 1 hepatotoxicity, 2.4% (*n* = 1) had grade 3 hepatoxicity requiring dose reduction, and 2.4% (*n* = 1) required permanent discontinuation of the treatment due to severe hepatotoxicity. Patient characteristics are shown in Table 1. None of the patients developed cardiotoxicity. When the QTcF values of the patients were compared, there was no significant difference at the beginning of day 14, and on the first day of the second and third cycle. The QTcF values of the patients and their comparison are shown in Table 2. The abnormalities in 24-h ECG rhythm Holter results before and in the third month of the treatment are shown in Table 3. None of the abnormalities were clinically meaningful or resulted in contraindications to ribociclib treatment. There was no statistically significant difference in rhythm Holter results before and after the treatment. EF (ejection fraction) of each patient was within normal limits, both at the beginning and in the third month. However, there were 16 patients with stage 1 diastolic dysfunction, and diastolic functions were normal in the other patients. In the echocardiography performed in the third month, it was seen that there was no increase in the number of patients with diastolic dysfunction. In addition, while the mean atrial diameter was 23.83 ± 16.83 mm in the first month, it was found to be 25.07 ± 17.15 mm in the third month echocardiography. No significant difference was found between the values.

**Table 1:** Patient characteristics

Age (mean ± SD)	**56.36 ± 12.74**
De novo metastatic *n* (%)	21 (50%)
BMI (mean ± SD)	28.36 ± 6.96
Smoking *n* (%)	11 (26.2%)
Endocrine therapy	
Fulvestrant *n* (%)	22
Aromatase ınhibitors *n* (%)	20
Comorbidities	
DM *n* (%)	6 (14.3%)
HT *n* (%)	13 (31%)
Neutropenia	
Grade 1 *n* (%)	4 (9.5%)
Grade 2 *n* (%)	22 (52.4%)
Grade 3 *n* (%)	13 (31.0%)
Grade 4 *n* (%)	0
Dose reduction (neutropenia related)	17 (40.5%)
Drug discontinuation (neutropenia related)	0
Hepatotoxicity	
Grade 1 *n* (%)	4 (9.5%)
Grade 2 *n* (%)	0
Grade 3 *n* (%)	1 (2.4%)
Grade 4 *n* (%)	1 (2.4%)
Dose reduction (hepatotoxicity related)	1 (2.4%)
Drug discontinuation (hepatotoxicity related)	1 (2.4%)

*DM* Diabetes mellitus, *HT* hypertension, *BMI* body mass ındex, *SD* standard deviation

**Table 2:** Comparison of QTc results during the first 3 cycles and between ECG

	QTc 1 (mean ± SD)	QTc 14 (mean ± SD)	QTc 2 (mean ± SD)	QTc 3 (mean ± SD)	*p*
ECG mSec (mean ± SD)	404.68 ± 26.56	412.45 ± 33.01	405.64 ± 23.27	405.96 ± 27.21	0.583

*SD* Standard deviation, *P* < 0.05: significant

**Table 3:** Twenty-four-hour rythym Holter results

	Normal	Rare PVC	Rare AES	PAF	Non-sustained VT
Holter 1	18	12	4	8	0
Holter 2	18	12	3	8	1

*PVC* Ventriculary extracystoles, *AES* atrial extracystoles, *PAF* paroxismal atrial fibrillation, *VT* ventriculary tachycardia

## Discussion

In this study, we evaluated the possible cardiac side effects of ribociclib with rhythm Holter and echocardiography in addition to routine ECG monitoring in the first 3 months of the ribociclib treatment. To our knowledge, this is the first study evaluating effects of ribociclib on cardiac rhythm with Holter ECG to detect clinical or subclinical rhythm changes. Our study showed no significant difference between the rhythm Holter and echocardiography results at the beginning of the treatment and at the third month.

CDK 4/6 inhibitors including ribociclib have become the new standard in the first-line treatment of hormone-positive HER2–negative metastatic breast cancer with their contribution to progression-free and overall survival [7, 15, 16]. Moreover, CDK 4/6 inhibitors have also been approved for use in adjuvant settings, showing a contribution to disease-free survival in the treatment of high-risk early and locally advanced breast cancer [17]. Although the general toxicity profile of CDK 4/6 inhibitors is manageable, the most common side effects have been reported as myelosuppression, gastrointestinal, and skin toxicities [7, 15, 16].When CDK 4/6 inhibitors were evaluated in terms of cardiac safety, it was observed that cardiovascular adverse events (CVAEs) were rare, but the most common events were QTc prolongation and venous thromboembolism. Furthermore, it was determined that only ribociclib caused QT prolongation, while other drugs in the group did not have such an effect [13]. Studies evaluating the QTc prolonging effect of palbociclib and abemaciclib showed no statistically significant effect on QTc with either drug. So, there is no any ECG monitoring recommendation for palbociclib and abemaciclib [18, 19].

It is of great importance that the safety of the drug and the easily manageable side-effect profile of the drug are as important as its effectiveness for the continuation of the treatment. Ribociclib, which stands out with its survival contribution among CDK 4/6 inhibitors and is preferred in NCCN 2023 guidelines, requires close ECG monitoring, especially in the first 3 months, due to its effect on QTc, unlike other drugs in this group [2].

According to the guidelines, it is recommended to calculate QTc by taking a baseline ECG before starting ribociclib treatment and to check QTc with ECG on the 14th day of treatment, and at the beginning of the second cycle. In general, it is not recommended to start treatment for those whose QTc is above 450 ms. In addition, if QTc rises above 480 ms during the treatment process, it is recommended to interrupt the treatment until it drops below 480 ms and then continue the treatment with the next lower dose. To detect electrolyte disorders that may cause QTc prolongation, blood tests should be performed at the beginning of treatment and regular intervals, and electrolyte levels should be adjusted if necessary. In addition, it should be checked whether patients are using other medications that may cause QTc prolongation. Closer monitoring is recommended in the presence of underlying cardiac arrhythmia or in patients using concomitant drugs that may cause QTc prolongation [2].

The safety and tolerability of various endocrine therapy combinations with ribociclib have been evaluated in recent years. MONALEESA-3 study reported QTcF prolongation resulted in discontinuation of the drug in 0.6% of patients receiving ribociclib + fulvestrant [20]. Furthermore, in the MONALEESA-2 study, QTcF interval was longer than 480 ms in 3.3% of the patients treated with ribociclib. An increase greater than 60 ms of QTcF interval was observed in 1.8% of the patients. Most of the patients were able to receive their treatment without dose escalation and interruption [15]. The total percent of the patients with dose interruption, reduction, or discontinuation was 1.4% in phase 3 trials of ribociclib [21]. Similarly, in our study, dose reduction or drug discontinuation due to cardiotoxicity was not required.

Furthermore, the real-world data of 1376 patients receiving CDK4/6 inhibitors showed that the frequency of cardiac adverse effects of CDK4/6 inhibitors was 24%, which was more than reported in previous studies [13]. The CVAEs were also not limited to QT prolongation, but there were various cardiac adverse effects, most commonly hypertension, heart failure, and atrial fibrillation [13]. The median time was 2.3 months for developing CVAEs. CVAE incidence was not different among the CDK4/6 inhibitors. Also, they showed that there was a slightly higher incidence of CVAEs among patients treated with CKD4/6 inhibitors compared with anthracyclines (*p* = 0.063). Different from this study, our patients had not experienced any clinically significant cardiac adverse effects in the first 3 month of the treatment. The fact that the mean age of the patients in the mentioned study is higher than that of our patient group and 40% of the patients were from Hispanic or Black races may be the reasons for this difference.

It is a limitation of our study that it followed up only a 3-month period, although the lack of change in cardiac rhythm Holter and echocardiography after 3 months of ribociclib use indicates the low risk for early cardiac toxicity of ribociclib. Since the median time until cardiac events were developed in the real-world data study was 2.3 months [13], we find it important for the cardiac safety of ribociclib that the rhythm Holter results at the third month showed no significant difference in our study. Another limitation was the small sample size, due to the exclusion of patients with known heart disease or using QT prolonging drugs. Additionally, measuring serum levels of cardiac biomarkers such as troponin and natriuretic peptides could provide a more comprehensive cardiac assessment.

## Conclusion

CDK4/6 inhibitors have shown promise in cancer treatment but may have cardiotoxic effects. In the present study, we could not detect any evidence of cardiotoxicity in addition to routine ECG monitoring with echocardiography and rhythm Holter test at the beginning and at the end of 3 months. So, we concluded that ribociclib had a low risk of early cardiotoxicity. Therefore, we agree that routine ECG monitoring is sufficient in patients using ribociclib. We may recommend further cardiac monitoring in patients with underlying cardiac disease and elderly patients.

It is of great importance for patients to be evaluated by a multidisciplinary team including oncologists, cardiologists, and pharmacologists, for protecting patients from possible CDK4/6 inhibitor cardiotoxicity and improving patients’ quality of life, while receiving the best possible cancer treatment.

## Data Availability

The data that support the findings of this study are available from the corresponding author, A.I.Y, upon reasonable request.
